# Minutes of the Austin District Medical Society

**Published:** 1890-04

**Authors:** T. J. Bennett

**Affiliations:** Sec’y.


### Society Notes.

### MINUTES OF THE AUSTIN DISTRICT MEDICAL SOCIETY.

The tenth quarterly meeting of this Society was held in Austin on the 27th ult., in the new hall fitted up jointly with the Travis County Medical Society. This was a very satisfactory meeting, there being thirty-three physicians and three medical students present.

The following papers were read and discussed: "The Surgical Treatment of Urinary Calculi," by Dr. Thos. D. Wooten; "Sudden Death in Labor," by Dr. L. H. Hardy; "Stone in the Bladder, with Lithotomy," by Dr. W. M. Cunningham; "La Grippe and Some of its Complications," by Dr. T. J. Bennett.

Dr. W. M. Cunningham read the following on the death of Dr.

J. F. Perry, of Red Rock, Bastrop county, which was ordered published, and spread upon the minutes, as a resolution from the Society:

"It becomes my sad duty to aanounce the death of Dr. J. F. Perry, of Cold Water neighborhood, Bastrop county. He joined our Society at the last meeting. On the 10th of March, 1890, near Red Rock, supported by the grace and love of the Savior of the world, he gently and peacefully passed away. He died from the effects of the deadful and insidious disease, malarial hæmaturia.

"In his life he was modest and unassuming, but earnest, energetic and brave. His purposes were for the faithful fulfillment of his duty as a husband, as a father, as a member of society, as a citizen of the country he loved, as a physician always ready to minister to the wants of the sick and distressed, and as a Christian humbling every act and deed of his life to the will of God who gave him life.

"He graduated in literature before he reached the age of maturity, from the renowned old school of Lebanon, Tenn. Shortly afterwards he began the study of medicine, and graduated from the medical school located at Nashville, at the early age of twenty-one. He was near fifty years of age at the time of his death, and had been in the practice of his profession thirty-five years. He leaves a wife and three children.

"In his death the country has lost a good citizen, Cold Water neighborhood a valuable physician and Christian gentleman, the sick and distressed a ready helper, and his family a kind husband and father.

"Let us, gentlemen, mingle our sadness and expressions of condolence with the tears of the bereaved family, and in the name of charity remind them that it is life to-day and to pass away to-morrow, and that life's greatest achievement is to be ready when the hour of death comes."

There were three new names admitted to membership, and one rejection.

The committee appointed at last meeting, Dr. J. W. McLaughlin, chairman, to confer with Dr. Wooten, President of the Board of Regents, relative to the Medical Department of the Texas University, asked for and was granted until the next meeting to make their report.

Dr. Bennett, chairman of the committee to select and fit up a permanent hall for the Society, reported that the work had been completed and paid for, but that he wanted until next meeting to present an itemized statement of expenses, etc. Time was granted.

The Society passed the following resolution:

*Resolved*, That the following card, handed in for opinion as to its ethics, is unethical where the physician does not limit himself to the diseases named in the card: "Doctor —— treats diseases of the rectum, urinary organs, and diseases of women."

The following delegates were elected to represent the Society at the Fort Worth meeting of the Texas State Medical Association: J. W. McLaughlin, F. R. Martin, W. M. Cunningham, Sam. Cunningham, E. Clark, T. D. Wooten, W. A. Morris S. E. Hudson, A. V. Doak, G. W. Christian, R. Adkisson, J. W. Carhart, R. S. Gregg, and R. M. Swearingen.

T. J. BENNETT, Sec'y.

